# Culinary Medicine: Needs and Strategies for Incorporating Nutrition into Medical Education in the United States

**DOI:** 10.1177/23821205241249379

**Published:** 2024-05-05

**Authors:** Olivia W. Thomas, Jo Marie Reilly, Nathan I. Wood, Jaclyn Albin

**Affiliations:** 1 Director of Nutrition Innovation and Implementation, 1836 Boston Medical Center, Boston, MA, USA; 2 Professor of Clinical Family Medicine and Population and Public Health Sciences, University of Southern California, Keck School of Medicine, Los Angeles, CA, USA; 3 Instructor of Medicine and Medical Education Fellow, 12228 Yale School of Medicine, New Haven, CT, USA; 4 Combined Internal Medicine and Pediatrics Residency; Culinary Medicine Program, University of Texas Southwestern Medical Center, Dallas, TX, USA

**Keywords:** Culinary medicine, medical school, nutrition, education, interprofessional

## Abstract

In the past decade, medical education has increasingly incorporated evidence-based lifestyle interventions as primary strategies for preventing and managing noncommunicable diseases. This shift embraces the growing recognition of the significant impact of lifestyle on health outcomes, driving diseases including obesity, diabetes, heart disease, and cancer. Now deemed “food is medicine” (FIM), diet-related interventions witnessed integration into healthcare systems and recognition in the United States’ White House Conference on Hunger, Nutrition, and Health in 2023. As FIM gains traction, investigating optimal strategies for team-based education becomes essential. Healthcare teams need the necessary knowledge and tools to effectively administer FIM services and collaborate across disciplines, ultimately enhancing disease prevention, chronic disease management, health quality, value, and overall wellness. Culinary medicine (CM), a vital component of FIM, bridges nutrition education, pragmatic culinary skills, and conventional strategies to improve chronic disease management. CM involves experiential learning, imparts practical skills, and encourages behavior change by addressing food-related determinants of health and promoting equitable access. Teaching kitchens serve as physical or virtual learning spaces and as a didactic and experiential method (skills lab), playing a crucial role by integrating culinary, lifestyle, integrative, and conventional medicine. A growing number of medical schools in the United States and globally offer CM education via diverse methods including interest groups, electives, and specialty tracks, encompassing didactic sessions, hands-on kitchen education, and virtual teaching methods. Given the rising demand for CM programs, this article aims to describe, map, and compare existing CM education types in medical education. It provides actionable recommendations for medical schools to establish and expand CM programs by fostering service-learning partnerships, clinical innovation, and interdisciplinary research. As FIM gains prominence, cultivating a robust foundation of educational strategies is vital to ensure seamless integration into both medical education and collaborative medical practice.

## Introduction

Evidence-based dietary and lifestyle behavior interventions are commonly accepted as a first-line step in medical practice to prevent and manage noncommunicable diseases such as obesity, diabetes, heart disease, cancer, and mental health disorders.^1,2^ Food and nutrition interventions, more recently referred to as “food is medicine” (FIM), are growing in popularity and are deployed in many health systems to address food and nutrition knowledge, food access, food preparation skills, and the food environment to support sustainable behavior change and improve health outcomes.^3^ According to the National Institutes of Health, FIM refers to five interventions and services: (1) medically tailored meals, (2) medically tailored and healthy food packages and groceries, (3) nutritious food referrals or vouchers, (4) prescriptions for nutritious groceries or produce prescriptions, and (5) culinary medicine (CM) and teaching programs. In addition to improved health outcomes, however, these services are also associated with decreases in healthcare utilization and cost savings across the healthcare continuum.^4–6^

In 2022, the U.S. House of Representatives passed House Resolution 1118,^7^ emphasizing the growing body of evidence supporting the positive impact of nutrition and the FIM movement on health. These congressional leaders aimed to inspire widespread adoption of FIM through educational innovation and clinical integration of services into traditional medical settings. To this end, the resolution was followed by a “Summit on Medical Education in Nutrition” hosted by the Accreditation Council for Graduate Medical Education, the Association of American Medical Colleges, and the American Association of Colleges of Osteopathic Medicine in March of 2023.^8^ In light of the growing support for multiple nutrition interventions and the FIM momentum and efforts, further research will determine the best practices to train collaborative healthcare teams with enhanced counseling skills and ability to direct these interventions. Teams must be equipped with the knowledge and tools necessary to galvanize FIM services and optimize interprofessional teams, ultimately improving the health and well-being of the population while reducing costs of care.^9–11^

A key component of FIM is CM, also referred to as culinary nutrition. CM helps translate nutrition education into practical skills and is used to improve chronic disease prevention, treatment, and management.^12–14^ Newman et al^15^ define CM education as translational food-based nutrition education offered through didactic and problem-based learning that may or may not include hands-on components (ie, cooking classes). Modifying food-related social determinants of health is essential to providing comprehensive FIM care. CM also addresses food insecurity and low-resourced kitchens by promoting the utilization of food assistance programs and related services.^4,16^

CM often includes experiential and hands-on learning to teach participants practical skills and enhance behavior change.^17^ Teaching kitchens are the physical or virtual spaces in which CM is practiced.^18^ These diverse spaces include industrial kitchens, community kitchens, university kitchens, and even home kitchens with both in-person and virtual applications.^19^ Teaching kitchens are positioned at the intersection of culinary, lifestyle, integrative, and conventional medicine as approachable and accessible learning laboratories for behavior change.^20^ During the COVID-19 pandemic, the necessity for distance learning drove further innovation in the field of CM. Many CM interventions have now been successfully adapted to virtual learning teaching kitchens.^19,21–23^

CM has also emerged as an effective method to teach practicing clinicians, medical trainees, and students about nutrition, diet, and practical food skills to improve both patient care and their own wellness. This type of experiential learning can be more effective than traditional medical education alone^24^ and is consistently well-received.^15^ CM curricula are dispensed via various modalities and range from traditional didactic learning in classrooms to hands-on CM training in teaching kitchens.^15,25,26^ Based on recent scoping reviews,^13,15,25^ more than 30 medical schools in the United States have already started CM programs, offering the foundation for CM implementation and scalability in medical education going forward. The programs described in the reviews, including their benefits and limitations, served as the foundation of this article's aim to identify the different methods for integrating CM into medical school education.

There is a growing need for foundational, evidence-based practice to help medical institutions establish CM programs globally. This includes building core interprofessional teams, supporting the expansion and enhancement of existing programs, and advancing service-learning community partnerships, clinical innovation, and research. This article aims to describe, map, and compare existing CM training offerings in medical education in the United States and to provide concrete recommendations for advancing the existing work.

## Body

### Approach

To better understand the adoption of and best practices for CM education in medical school, a group of subject matter experts reviewed two recent scoping reviews^15,25^ to identify types of education and barriers and facilitators for implementation. Articles that were included in the scoping reviews were thematically coded and organized into emergent themes. Consensus was established among experts and the themes were then used to describe and compare types of CM education and to make recommendations for creating new programs or scaling existing programs.

### Types of CM Training in Medical Education

The application of CM in medical education aims to teach nutrition principles relevant to patient care through cost-effective and scalable methods while promoting skills applicable to personal dietary wellness. This article describes six education types with varying approaches and intensities including (1) curricular enhancement, (2) service-learning, (3) extracurricular interest groups, (4) electives, (5) curricular integration, and (6) specialty tracks (Table 1). Each type can be tailored to the needs of a given program or institution.

**Table 1.** Types of culinary medicine education in United States medical schools.

MODALITY	DESCRIPTION	PROGRAM EXAMPLES
Curricular enhancement	Incorporate CM concepts, literature, and videos into existing education via short, in-class lessons, discussion, or assignments. Enhancements are often included at the discretion of the course lecturer and are not formally integrated into curricula.	Penn State College of Medicine vertically integrates a collaborative cook-off competition in a basic science course through a constructivist educational model.^27^
Service-learning	Voluntary experience to work hands-on with the community through food-related service. Experiences are facilitated through community and/or clinical partnerships and may or may not have didactic components.	The Keck School of Medicine of the University of Southern California offers a community nutrition-based service-learning course for its primary care track pipeline students.^28^ University of Alabama at Birmingham engaged medical students in Cooking Healthily on a Penny, a program that delivers community-based cooking demonstrations while building student counseling confidence and awareness of how social determinants of health impact food and nutrition choices.^29^ The University of Arizona offers service-learning hours that include a variety of nutrition education and cooking demonstrations in the community for medical students and other health professional students in a university and statewide collaboration between the health sciences, nutrition, university libraries and cooperative extension in the agricultural college. These activities occur with a variety of community partners including federally qualified community health centers, school districts, tribal nations, farmer's markets, food banks and medically tailored meal programs with special populations.^30^
Extracurricular activities and interest groups	Voluntary learning opportunities outside of standard education that may or may not be recognized by the institution and are often facilitated through community partnerships, interprofessional teams, student groups, affinity groups, and/or professional groups.	Boston University's Chobanian and Avedisian School of Medicine offers a voluntary six-week extracurricular CM course in partnership with a local health system. The course is offered as part of a student-led nutrition interest group.^31^ Wayne State University School of Medicine has historically offered a series of voluntary, student-led culinary nutrition classes for interested first-year medical students. Funding was provided in partnership with a student-led interest group and the medical school's alumni association.^32^ Baylor College of Medicine offers a series of voluntary sessions incorporating both didactic learning and hands-on cooking experiences called “Choosing Healthy, Eating Fresh” to its second-year medical students. The initiative is organized and offered by a student-led medical student organization.^33^ The American College of Lifestyle Medicine supports Lifestyle Medicine Interest Groups with small grants to support nourishing food options at lifestyle-focused events, and many groups build CM content and activities into their programs.^34^
Electives	Voluntary classes outside of standard education that are formally recognized by the institution and are often led by faculty members (normally as a collateral duty) and sometimes facilitated in partnership with community programs and/or interprofessional teams.	University of Texas Southwestern offers a CM elective to medical students, physician assistant students, and other health professional graduate students at an on-campus kitchen. The elective includes monthly hands-on classes and is co-led by a physician and registered dietitian nutritionist with culinary training.^15,35^ Keck School of Medicine of the University of Southern California partnered with the Los Angeles General Hospital Wellness Center Kitchen to offer a voluntary hands-on preclinical culinary and nutrition elective for medical students.^36^ Northwestern University Feinberg School of Medicine, in partnership with the Osher Center for Integrative Health, offers a CM elective for medical students on the Integrative Medicine track.^37^ Touro University Nevada College of Osteopathic Medicine offers an experiential CM elective for medical students as an adjunct to its traditional curriculum.^38^ The University of Arizona College of Medicine Phoenix offers a culinary medicine elective for fourth year medical students open to all VSLO students nationally that includes cooking demonstrations, didactic sessions, and experiential learning activities with local chefs, nutrition faculty in the university dietetics program, cooperative extension agents, local farmers, local schools, and medical school faculty.^30^
Curricular integration	Required lessons that are embedded within standard medical education to enhance learning and provide awareness to entire cohorts which are often interprofessional. Curricula are often a combination of didactic and hands-on learning.	First- and second-year medical students at Tulane University School of Medicine participate in required culinary medicine classes as part of their Foundations in Medicine coursework.^39^ Medical students at University Integrative Health (a community of Maryland School of Medicine) are required to take a first-year experience that is a component of the core medical student curriculum. The course includes didactic lectures and cooking lessons.^40^ Virginia Tech Carilion School of Medicine integrates CM as an educational strategy within their existing interprofessional education course.^41^
Specialty track	Formal training integrated into medical education through specialized tracks. Experiences are either vertically integrated over multiple years or are multisession intensive learning.	At West Virginia University School of Medicine, medical students can apply to be part of a robust, four-year Culinary and Lifestyle Medicine Track that equips them through online modules, teaching kitchen experiences, and patient care application to be eligible to sit for the Culinary Medicine Certification (CCMS) exam after completing medical school.^42^ At the University of Arizona College of Medicine Phoenix, medical students can apply to be part of a four-year Wellness, Integrative Medicine, and Nutrition Certificate of Distinction track which includes experiential and service learning in the community, seminars, online modules, teaching kitchen experiences in a variety of community settings, clinical case preparation, and seminars by experts.^30^

### Curricular Enhancement

Curricular enhancements refer to incorporating CM concepts into lectures through classroom demonstrations, assignments, literature reviews, and discussion. For example, an anatomy lecture on the gut microbiome may include brief teaching on the application of nutrition recommendations to food choices, an assignment to prepare a meal with probiotics, and a subsequent online forum or in-class discussion. These types of enhancements are often included at the discretion of the course lecturer and are not formally integrated into curricula. As such, there are minimal published examples of curricular enhancements or their impact on medical education.^27^

### Extracurricular Activities and Interest Groups

Extracurricular activities are learning opportunities outside of formal education and, sometimes, outside of the organization itself. CM extracurricular activities have been described as workshops, monthly meetings, or training sessions that are not part of a formal elective.^29,41^ Extracurricular activities include professional conference attendance and student interest group participation. For example, medical students can choose to attend conferences like “Healthy Kitchens, Healthy Lives” organized by the Harvard School of Public Health and the Culinary Institute of America^43^ or join national interest groups like the American College of Lifestyle Medicine's Medical Student Members Interest Group.^34^ In addition to national efforts, locally applied versions of these interest groups^31–33^ also exist within individual medical schools and communities and sometimes offer dedicated CM experiences.

## Electives

CM education is often offered as an elective recognized by an academic center and includes didactic learning through online or in-person lectures and hands-on culinary experiences.^15,25^ Some electives also include case-based learning in groups and class assignments. Although funding information is limited, one scoping review suggests that most electives are funded through medical schools, the dean's office, philanthropy, or various grants.^15^ Funding is necessary for the food and supplies needed for hands-on training in addition to maintenance of learning spaces, particularly if a physical teaching kitchen is utilized. Instructors are often volunteers and support programs through collateral duties. Most programs use interprofessional teams as course instructors^15^ with many examples including a registered dietitian nutritionist as the nutrition expert.^16,35^ There is little standardization of elective goals and objectives, layout, duration, type of instructor(s), and assessment tools.^15^ Some programs use existing evidence-based curricula, including the *Health Meets Food* curriculum and American College of Lifestyle Medicine's “Culinary Medicine Curriculum.”^44,45^

### Service-Learning

Service-learning opportunities in CM are voluntary or curricular integration experiences facilitated through community organizations or clinical partnerships. The Liaison Committee on Medical Education defines service-learning as “a structured learning experience that combines community service with preparation and reflection. Students engaged in service-learning provide community service in response to community-identified concerns and learn about the context in which service is provided, the connection between their service and their academic coursework, and their roles as citizens and professionals.”^46^ Although service-learning programs are common in medical education, programs with a focus on CM are limited.^28,30^ They typically include opportunities to partner students with local community organizations to provide patient nutrition education as the “hands-on” application of their didactic nutrition education. Service-learning also promotes exploring the intersection of nutrition and food-based education with other social determinants of health, such as understanding barriers to accessing nourishing food.

### Curricular Integration

Curricular integration refers to CM concepts or experiences embedded in standard medical education curricula that reach the entire student body. Given its breadth, curricular integration is often didactic, and lectures or discussions may be delivered online or in person. However, some medical schools require hands-on cooking and applied CM experiences via service-learning.^40^ This horizontal integration provides a broad awareness of CM while cultivating champions who may go on to seek further training.

### Specialty track

Specialty tracks refer to formally integrated, multisession CM training experiences that are offered through specialized tracks and sometimes take place over multiple years. This type of vertical integration results in intensive learning that results in CM champions, practitioners, and experts. At least one example of these specialty tracks prepares medical student participants to become certified in CM after graduation.^42^

### Comparison of CM Training Approaches in Medical Education

**Figure 1.**
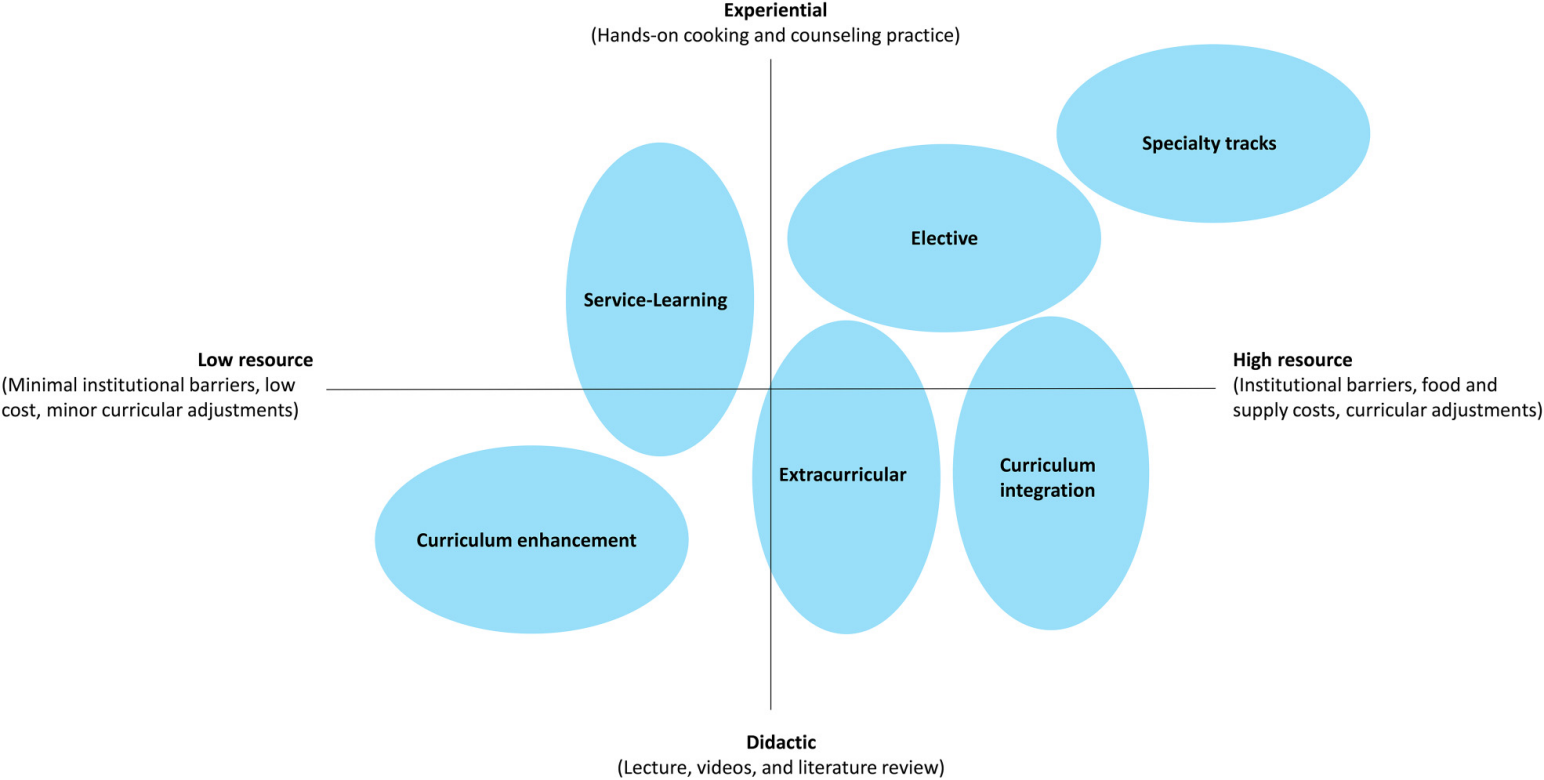
Culinary medicine curricular types by resource investment (attached separately).

These six education types are compared based on learning approaches (didactic vs experiential) and available resources needed to implement them (low vs high) (see Figure 1). Within each approach, different methods and different levels of resource investment are needed; thus, each approach represents an area on the graph rather than a single point. There are opportunities and costs with each CM modality selected. Offering multiple options in the same institution, if possible, is ideal. Curricular enhancements are typically low-resource options that allow an introduction to CM without budgetary needs or major curricular adjustments. In fact, most examples of enhancements are didactic or demonstrative with limited opportunities for students to participate in hands-on or experiential learning. Service learning may also require reduced resources as this type of education is often offered through partnerships with already existing external groups and programs.

A high-resource CM application includes electives offered outside of core medical education. Electives in CM generally promote some didactic training but focus more on hands-on experiences. As such, these electives often require a significant investment in several resource domains, including time, space, food acquisition, and facilitator expertise. Extracurricular activities tend to be more focused on small group or individual experiences yet still require time and funding for students to engage in the learning and training. Resource availability varies from institution to institution and individual institutions can optimize success based on a local needs assessment, student demand, and available support across key resource domains. Online and video-based programs, by utilizing existing curricula or partnering with other institutions, may be less resource-intensive, as they do not require physical spaces or continuous content development. However, electives offered in physical teaching kitchen spaces with opportunities for hands-on culinary experiences, while rich in experiential learning, may be more resource-intensive.

High-resource CM training offers greater breadth and depth for a larger cross-section of medical trainees. However, curricular integration requires extensive planning, determination of appropriate integration space and timing, coordination with institutional education committees, and alignment with competencies. Although resource-intensive, integrating CM training into core medical education ensures global awareness of both the important role of food in health and its application to patient care. It also builds future FIM leaders for the rapidly growing field at large.

Alternatively, specialty tracks are an example of more intensive CM training that results in champions, experts, and future certified CM practitioners. At present, only one medical school offers a CM track that provides vertical opportunities to learn and practice skills while providing documented CM specialization training on academic transcripts.^42^

For each of these CM education modalities, there exists the opportunity to focus wholly or in part on virtual CM training. For instance, the completely virtual, asynchronous “Nutrition in Medicine” coursework that was offered by the University of North Carolina at Chapel Hill has been utilized by dozens of medical schools since its inception over 20 years ago in a variety of educational contexts.^47^ With community-based CM sessions increasingly being held online,^23^ there exist opportunities for medical trainees to engage in virtual service-learning by participating in these sessions. Extracurricular activities organized through student organizations and interest groups often utilize virtual teaching kitchens, such as the example of Baylor's Choosing Healthy, Eating Fresh program. CM electives, similarly, may use virtual didactics and kitchens to reach more students—in some instances, allowing for multiinstitutional reach.^21^

Like in-person CM education modalities, virtual CM offerings are broad in intensity and type of learning method utilized, as noted by the wide range of resources and experiences depicted in Figure 1. With a tripod and smartphone, a virtual teaching kitchen can be set up in an instructor's home—a low-resource approach. In contrast, physical teaching kitchens can be retrofitted with professional quality audio/video streaming equipment to conduct classes in virtual spaces, representing a high-resource approach. Asynchronous lectures can be recorded using video-conferencing software and dispensed to learners via online portals or an institution's learning management system—a didactic virtual approach to CM education. Virtual teaching kitchens, with participants communally cooking together on a video conference call from their respective home kitchens, exemplify a more experiential approach to CM education.

## Discussion

Medical education currently faces multifaceted challenges as institutions and educators assess both the optimal curricula and delivery modalities necessary to prepare learners for the breadth, depth, and scope of clinical practice and the evolving complexity of the U.S. healthcare system. With the worsening chronic disease pandemic closely linked to lifestyle behaviors, many educators are integrating CM and lifestyle medicine into medical education. To aid in this effort, this article defines the multiple modalities in which CM is taught in medical institutions. It describes the benefits and challenges of each method. Many institutions employ a combination of teaching modalities that provide both horizontal and vertical paths for more advanced CM training.

Curriculum enhancement provides opportunities for educators to adopt CM training practices with minimal need for curricular adaptation or funding acquisition. Educators can consider incorporating relevant literature or food-based activities into existing content, including those that are self-directed by learners and promote personal student wellness. Service-learning offers community partnership, professional development, and the opportunity for students to engage in the “hands-on application” of their CM didactic curriculum in a community-based setting with rich practical learning. However, service-learning requires strategic, bidirectional, and longitudinal partnerships with clinical and/or community organizations. While this type of CM learning may require fewer resources from the medical institution, a strong partnership for implementation is critical.

Electives, service learning, and extracurricular activities can offer CM training within an institution.^15,25,35–38^ Activities are typically delivered by interprofessional teams either as collateral duties or as compensated experts and facilitators. Strategies to reduce required resources have been documented, including adopting existing curricula like *Health meets Food*,^15^ offering pre-recorded lectures and virtual hands-on experiences in a student's home kitchen,^37,48^ and incorporating an interprofessional team with emphasis on the inclusion of registered dietitian nutritionists.^49,50^

Curricular integration requires CM training as part of the core medical curriculum^40^ and increases CM exposure to a broader scope of medical students. However, formally integrating new content into medical education, even at a single institution, is an intensive task. New content needs to be reviewed and vetted by curricular committees and medical education oversight boards. Planning also necessitates the coordination of key competencies across several domains. Thus, integrating CM curricular training across the four years of medical school is an ideal but rigorous and labor-intensive endeavor. Additionally, due to limited resource constraints, most institutions provide CM training outside of physical teaching kitchen spaces, necessitating more didactic and/or virtual learning.

Specialized tracks are a method for creating CM champions and practitioners who are equipped to practice promptly after the completion of formal training. Students learn and practice skills over years and/or multiple courses. Academic tracks are an intensive way for individual institutions to provide specialized training in CM and can potentially serve as a program recruitment tool.

Virtual CM training offers unprecedented flexibility and opportunity for increasing the reach of CM in medical education. Any of the described educational modalities can be dispensed virtually and, when necessary, accomplished using very few resources. Asynchronous and online lectures and virtual teaching kitchens are increasingly being used to offer nutrition education and hands-on CM training to students.

## Limitations

The variability in medical schools’ CM training options and resource availability limits the standardization of CM's content, learning objectives, course structure, instructors, target audience, and curricular assessment. This makes a rigorous analysis of the various CM training options difficult. Due to limited evidence, difficulty in developing CM educational best practices persists. Additionally, this article's analysis is limited to medical education examples and methods in the United States based on the currently available scoping reviews, perhaps limiting its applicability to a global audience, though some research suggests medical students in the United States and France have similar perceptions and needs regarding nutrition education.^51^

## Future direction

The recent U.S. House Resolution 1118 calls for the inclusion of food and nutrition education in medical training and offers an opportunity to explore innovative nutrition education methods. Among these approaches, CM has emerged as a feasible, popular, and effective method to provide medical students with practical food skills and improve patient care and wellness.^15,25^ Despite its growing popularity, best practices for CM education are undefined. This article aims to both describe and compare the emerging types of CM education based on the learning styles and resources needed to establish best practices, forging paths for new and existing CM programs. Considering the need for sustainability and longevity of these efforts beyond the current trends, it is critical to develop formal nutrition educational requirements for medical students, including hands-on experiences promoting nutrition and culinary literacy, like CM. Integration of foundational nutrition and culinary curricula even earlier in the educational continuum also has value and should be explored based on successful experiences at the university level in the United States and Germany.^52,53^ Furthermore, the integration of nutrition and sustainability content in the education of global culinary experts and chefs enhances opportunities for interprofessional collaboration.^54^

Incorporating nutrition, and more specifically CM, into core medical education is critical for patient care and to transition diet and lifestyle interventions from tertiary to primary care prevention. Furthermore, training medical students in CM helps enhance their interprofessional (IP) knowledge and skills. The latter are needed for collaborative medical care practice and to allow teams to work optimally, to reduce clinician burnout, and to enhance patient resources. Additionally, cultivating CM IP clinical experts provides more opportunities for specialization and professional competitive advantage. As medical educators develop alignment on the necessary competencies for nutrition in practice, the value of a broad-based nutrition education toolkit supports the needs of diverse training programs. As individual medical schools consider CM best practices, they should consider starting with lower-resourced options. These opportunities include service-learning, extracurricular, and elective CM programs, some, or all of which may be offered virtually. Medical schools’ optimal goal would be to integrate experiential CM into core curricula while also offering specialized tracks and certificates for students seeking advanced expertise.

## Conclusion

As clinicians respond to the growing need to address chronic disease management with prevention and lifestyle modification, medical educators must provide nutrition education to their trainees to better prepare them for the evolving healthcare landscape. Identifying nutrition educational best practices and incorporating CM and FIM training into core medical curricula is essential. Lastly, prioritizing opportunities for standardization and assessment—including requiring nutrition testing on medical board exams—will ensure that future physicians are better equipped to meet the nutritional needs of their patients and work effectively on interprofessional medical teams.
